# The effects of self-efficacy and social support on behavior problems in 8~18 years old children with malignant tumors

**DOI:** 10.1371/journal.pone.0236648

**Published:** 2020-07-31

**Authors:** Qian Liu, Lin Mo, Xianqiao Huang, Lu Yu, Yang Liu

**Affiliations:** 1 The Academy of Pediatrics of Chongqing Medical University, Chongqing, P.R China; 2 Children's Hospital of Chongqing Medical University, Chongqing, P.R China; 3 Ministry of Education Key Laboratory of Child Development and Disorders, Chongqing, P.R China; 4 National Clinical Research Center for Child Health and Disorders, Chongqing, P.R China; 5 China International Science and Technology Cooperation base of Child Development and Critical Disorders, Chongqing, P.R China; 6 Chongqing Key Laboratory of Pediatrics, Chongqing, P.R China; 7 Department of VIP Outpatient, Chongqing Medical University Affiliated Children's Hospital, Chongqing, P.R China; 8 Department of Hematology, Chongqing Medical University Affiliated Children's Hospital, Chongqing, P.R China; Gachon University Gil Medical Center, REPUBLIC OF KOREA

## Abstract

**Objective:**

To explore the influence factors of behavior problems of 8~18 years old children with malignant tumors in the treatment period, as well as the mediating effects of social support self-efficacy and post-traumatic growth.

**Methods:**

From May 2019 to October 2019, 160 children with malignant tumors during the treatment were selected through convenience sampling method, and were investigated via the General Self-Efficacy Scale, Social Support Questionnaire, Post-Traumatic Growth Scale and Conners’ Parent Symptom Questionnaire. Structural equation model was established on the basis of survey results.

**Results:**

The total detection rate of behavior problems in 8~18 years old children with malignant tumors was 10.6%. The structural equation models indicated that boys had more behavior problems than girls, self-efficacy, social support and post-traumatic growth can directly affect the behavior problems of 8~18 years old children with malignant tumors in the treatment period, and the standardized total effect of self-efficacy in both of the first model and the second model was the largest. Self-efficacy is also able to indirectly and negatively predict the behavior problems based on social support or post-traumatic growth. Social support can directly affect behavior problems or indirectly predict behavior problems through self-efficacy and post-traumatic growth. After 2000 bootstrap tests, the mediating effects of social support self-efficacy and post-traumatic growth were confirmed.

**Conclusion:**

Reduced total detection rate of behavior problems suggests that targeted interventions in recent years may be effective. Interventions focused on improving self-efficacy, social support and post-traumatic growth may lessen behavior problems of children with malignant tumors in the treatment period.

## 1. Introduction

As the most common problem in children's mental health, child behavior problem refers to the abnormal psychological behavior that the severity and duration exceed the normal range allowed by the corresponding age, such as attacks, violation of discipline, social withdrawal and so on [1]. Children with malignant tumors are in a special period of growth and development, facing long-term hospitalization and long treatment cycle, which easily affects the development of cognition and behavior, and leads to social withdrawal, poor communication and violation of discipline [2–4]. Additionally, the discrimination from society and excessive family care make the behavior problems of children with malignant tumors prominent, which has become a major public health problem that can not be ignored.

Self-efficacy is an individual's speculation and judgment on whether he or she has the ability to complete a certain behavior, which can reflect an individual's belief in taking appropriate actions to face environmental challenges [5]. Although no research has directly confirmed the relationship between self-efficacy and behavior problems of children with malignant tumors, some studies showed that parental self-efficacy predicted children's behavior problems [6] and high self-efficacy was conducive to taking healthy behaviors [7]. We can assume that individuals with low self-efficacy have no confidence in success when facing setbacks and challenges, meaning they may have some behavior problems such as withdrawal and discouragement. In addition, the behavior formula of Lewin shows that individual behavior is the result of the interaction between individuals and their situation [8]. Self-efficacy, as an important internal factor, may be one of the influencing factors of behavior problems of children with malignant tumors.

Post-traumatic growth (PTG) is a positive psychological change obtained after struggling with traumatic events or situations [9] and brings positive changes to individuals, including strengthening the sense of responsibility, improving interpersonal relationships and helping others [10–11]. No study has proved that PTG can affect the behavior problems of children with malignant tumors, but a small number of researches have shown that positive PTG can predict prosocial behavior [12–13] and promote patients with malignant tumors to adopt healthy behavior [14]. Additionally, PTG can reduce the risk of suicide in individuals who have experienced traumatic life events [15].

Social support theory intuitively points out that social support is an important protective factor of mental health. Psychological conflict and behavior problems are more likely to happen when individuals cannot achieve or achieve less external support, which shows that social support of children is closely related to their behavior problems [16]. Previous studies demonstrated that social support had a buffering effect on stress [17] and was a protective factor for adolescents’ behavior problems [18–19]. In addition, the previous findings suggested that social support could directly predict self-efficacy and indirectly affect self-management behaviors through self-efficacy [20].

At present, the published studies only reported the effects of gender, illness time, family rearing style, family relationship and coping style on the behavior problems of children with malignant tumors [21–22]. Therefore, this study intends to actively explore the effects of individual and environmental factors such as self-efficacy, PTG and social support on behavior problems, in order to provide a new perspective for preventing or reducing the occurrence of behavior problems in children with malignant tumors during treatment.

## 2. Materials and methods

### 2.1. Design

This study was a cross-sectional descriptive study in which convenience sampling was adopted to survey the children with malignant tumors. The Ethics Committee of Children’s Hospital of Chongqing Medical University approved this study. Besides, the study has obtained the consent of the participants’ parents or legal guardians who signed the informed consent form.

### 2.2. Participants

Children with malignant tumors was recruited from the Children’s Hospital of Chongqing Medical University from May 1, 2019 to October 1, 2019. Inclusion criteria were: children with malignant tumors who have started treatment; 8 to 18 years old (children aged 8 years old and above have the ability to self-report feelings [23]); able to comprehend and communicate; signed informed consent obtained from the patient’s parents or legal guardians. Patients, who were unable to understand the questionnaires, who suffered from severe organic or mental diseases, who were the recurrence of malignant tumors, or who refused to be investigated were excluded from this study.

This study included 19 variables, including age range, gender, type of disease, living area, affirmation and support, company and intimacy, satisfaction, conflict and punishment, self-efficacy, relationship with others, new possibilities, personal strength enhancement, mental change, appreciation of life, conduct problem, learning problem, psychosomatic disorders, impulsivity-hyperactivity and anxiety. According to sample size estimation method of Kendall [24], the sample size of multivariate analysis is 5 to 10 times of the number of variables, then the calculated sample size is 95 to 190 cases. After considering the loss of follow-up rate of 10%, the final sample size is 105 to 209 cases.

A total of 166 patients were selected according to the standards, however, two patients later changed their mind not to fill in the questionnaires, one patient lacked the score of PTG and three patients lacked the score of social support. All the data of these six people were not included in the analysis and 160 valid questionnaires were recovered, with an effective recovery rate of 96.4%. Of the 160 participants, 123 patients only received chemotherapy, and 37 patients received surgery and chemotherapy.

### 2.3. Instruments

Demographic and clinical characteristics included age ranges (8~12 years old = 0, 13~18 years old = 1), gender (boy = 0, girl = 1), type of disease (leukemia = 0, lymphoma = 1, sarcoma = 2, others = 3) and living areas (rural = 0, urban = 1).

The following questionnaires were used to gather information in this study.

General self-efficacy was measured by the General Self-Efficacy Scale [25] with 10 items. Each item is scored from 0 (quite wrong) to 3 (quite right). The total score ranges from 0~30; higher total score indicates higher sense of self-efficacy. Based on the score index, the level of self-efficacy was divided into three grades: high (80% and above), medium (60% ~ 80%) and low (< 60%).

Social network questionnaire [26] is a 5-point scale from 1 (never) to 5 (almost), which was modified and used to measure social support. This scale has 36 items, measuring affirmation and support, company and intimacy, satisfaction, conflict and punishment. The higher the total score is, the heavier the degree is.

Post-traumatic growth (PTG) was calculated following the Post-traumatic Growth Assessment Scale [27], which is a 21-item scale with 5 dimensions. Each item is scored from 0 (no change at all) to 5 (change a lot) and summary scores range from 0 to 105. Higher scores show more PTG.

Child behavior problem was measured by Parent Symptom Questionnaire (PSQ) [28] with 48 items, and each item is scored on a 4-point scale from 0 (never) to 3 (quite a lot). This questionnaire is filled in by child’s father or mother and higher scores indicate that the behavior problems are more serious. The instrument assesses and measures six dimensions of child behavior problem. However, only conduct problems, learning problems, psychosomatic disorders, impulsive-hyperactive and anxiety were included in the study. The standard score is the sum of the scores of each dimension divided by the number of items. We referred to the norms of the PSQ in Chinese Urban Children [29], and any factor scored greater than ($\overset{-}{X}$+ 2SD) was considered as abnormal behavior.

### 2.4. Data collection

The research staff were fully trained for 7 days before surveying. The questionnaires were completed by the parents and children on their own in the presence of the research staff. To ensure the quality of the survey, another research staff checked the questionnaires. During or after data collection, all authors had access to information that could identify individual participants.

### 2.5. Statistical analyses

The mean±standard deviation ($\overset{-}{X}$±SD) was used to express the measurement data, the percentage (%) was used to express the counting data. The standard scores of PSQ were used for preliminary descriptive and group comparisons analyses. Independent-sample t-test and one-way ANOVA were used for statistical analysis. Chi-squre(χ²) test was used between groups. All tests were two-sided and statistical significance was defined as p < .05. Bonferroni method was used to adjust the test level for one-way ANOVA. Bivariate analyses of self-efficacy, social support, PTG and behavior problem were performed using Pearson correlations with SPSS 22.0. The structural equation model was conducted with AMOS 22.0 using maximum standard likehood estimation and the raw scores of all variables have been used in structual equation model and bivariate analyses. Our model was based on three latent variables, social support, PTG and behavior problem, and one observed variable, self-efficacy. We evaluated the model fit using the chi-squared statistic with the normed chi-square (χ^2^/df), the Root Mean Square Error of Approximation (RMSEA), the Comparative Fit Index (CFI), the adjusted Goodness-of-Fit Index (AGFI), the Goodness-of-Fit Index (GFI) and the Normed Fit Index (NFI). The estimate of indirect effect was examined using Mplus 8.3.

## 3. Results

### 3.1. Sample characteristics and behavior problems

The sample characteristics and behavior problems of 160 patients were showed in Table 1.

**Table 1:** Sample characteristics and behavior problems (N = 160).

Characteristics	n (%)	$\overset{-}{X}$±SD
Gender		
boy	93(58.1)	
girl	67(41.9)	
Age range (years)		
8~12	107(66.9)	
13~18	53(33.1)	
Type of disease		
leukemia	94(58.8)	
lymphoma	27(16.9)	
sarcoma	17(10.6)	
others	22(13.8)	
Living area		
rural	113(70.6)	
urban	47(29.4)	
Self-efficacy		14.3±4.2
low-level	124(77.5)	12.6±2.8
medium-level	32(20.0)	19.6±1.9
high-level	4(2.5)	24.5±0.6
Social support		
affirmation and support		142.5±29.4
company and intimacy		98.8±21.5
satisfaction		62.1±11.2
conflict and publishment		82.0±19.0
Post-traumatic growth		41.6±15.3
relationship with others		14.1±5.3
new possibilities		9.3±4.8
personal strength enhancement		8.0±4.0
mental change		2.8±2.0
appreciation of life		7.8±3.3
Behavior problem		
conduct problems		.53±.34
learning problems		.79±.51
psychosomatic disorders		.41±.35
impulsivity-hyperactivity		.53±.48
anxiety		.49±.42

“Others” includes immature teratoma, dysgerminoma, yolk sac tumour of the ovary, neuroblastoma and hepatoblastoma.

As reported in S2 Table, the scores of learning problem dimension and anxiety dimension of behavior problem were significant different in different gender (both *P* < .05) and both of them were higher in boys. The scores of company and intimacy dimension of social support were significant different in different age ranges (*P* < .05), which was higher in 8~12 years old children with malignant tumors. In addition, the scores of conflict and punishment dimension were significant different in different age ranges and different living areas (both *P* < .05), and the scores of children aged 13~18 years and living in urban areas were higher. In all dimensions of PTG, it was only found that the scores of relationship with others were significant different in different living areas (*P* < .01), and children living in rural areas had higher scores on the dimension of relationship with others.

Among 160 children with malignant tumors, the total detection rate of behavior problem was 10.6% (17/160). In addition, the detection rate of conduct problem, learning problem, psychosomatic disorders, impulsivity-hyperactivity and anxiety were 4.4% (7/160), 3.8% (6/160), 3.1% (5/160), 3.1% (5/160) and 7.5% (12/160), respectively.

### 3.2. Bivariate analyses

The results of bivariate analyses was shown in Table 2. Self-efficacy, all dimensions of PTG, affirmation and support, company and intimacy were negatively correlated with child behavior problems. There was a significant negative correlation between satisfaction and conduct problems, and conflict and punishment was positively correlated with conduct problems.

**Table 2:** Correlation between observed indicators of latent variables (N = 160).

Variables	Correlations between Variables														
	1	2	3	4	5	6	7	8	9	10	11	12	13	14	15
1 Self-efficacy	1	—	—	—	—	—	—	—	—	—	—	—	—	—	—
Social support															
2 affirmation and support	.30^a^	1	—	—	—	—	—	—	—	—	—	—	—	—	—
3 company and intimacy	.31^a^	.80^a^	1	—	—	—	—	—	—	—	—	—	—	—	—
4 satisfaction	.13	.63^a^	.61^a^	1	—	—	—	—	—	—	—	—	—	—	—
5 conflict and publishment	-.10	-.54^a^	-.59^a^	-.59^a^	1	—	—	—	—	—	—	—	—	—	—
Post-traumatic growth															
6 relationships with others	.29^a^	.25^a^	.21^a^	.11	-.11	1	—	—	—	—	—	—	—	—	—
7 new possibilities	.45^a^	.20^b^	.21^a^	.10	-.11	.61^a^	1	—	—	—	—	—	—	—	—
8 personal strength enhancement	.33^a^	.18^b^	.20^b^	.10	.05	.63^a^	.72^a^	1	—	—	—	—	—	—	—
9 mental change	.28^a^	.06	-.01	-.11	.01	.34^a^	.48^a^	.38^a^	1	—	—	—	—	—	—
10 appreciation of life	.24^a^	.05	.00	.02	-.02	.46^a^	.46^a^	.48^a^	.27^a^	1	—	—	—	—	—
Behavior problem															
11 conduct problems	-.45^a^	-.38^a^	-.34^a^	-.21^a^	.19^b^	-.20^a^	-.33^a^	-.26^a^	-.25^a^	-.21^a^	1	—	—	—	—
12 learning problems	-.28^a^	-.24^a^	.18^b^	-.15	.06	-.27^a^	-.24^a^	-.29^a^	-.17^b^	-.29^a^	.46^a^	1	—	—	—
13 Psychosomatic disorders	-.48^a^	-.33^a^	-.27^a^	-.15	.11	-.27^a^	-.34^a^	-.28^a^	-.29^a^	-.25^a^	.82^a^	.58^a^	1	—	—
14 impulsivity-hyperactivity	-.49^a^	-.17^a^	-.16^b^	-.08	.08	-.27^a^	-.39^a^	-.27^a^	-.25^a^	-.29^a^	.51^a^	.30^a^	.62^a^	1	—
15 anxiety	-.47^a^	-.33^a^	-.28^a^	-.14	.12	-.30^a^	-.39^a^	-.36^a^	-.29^a^	-.28^a^	.86^a^	.70^a^	.85^a^	.60^a^	1

^*a*^: *P* < .01;

^*b*^: *P* < .05.

### 3.3. Structural equation model

**Fig 1:**
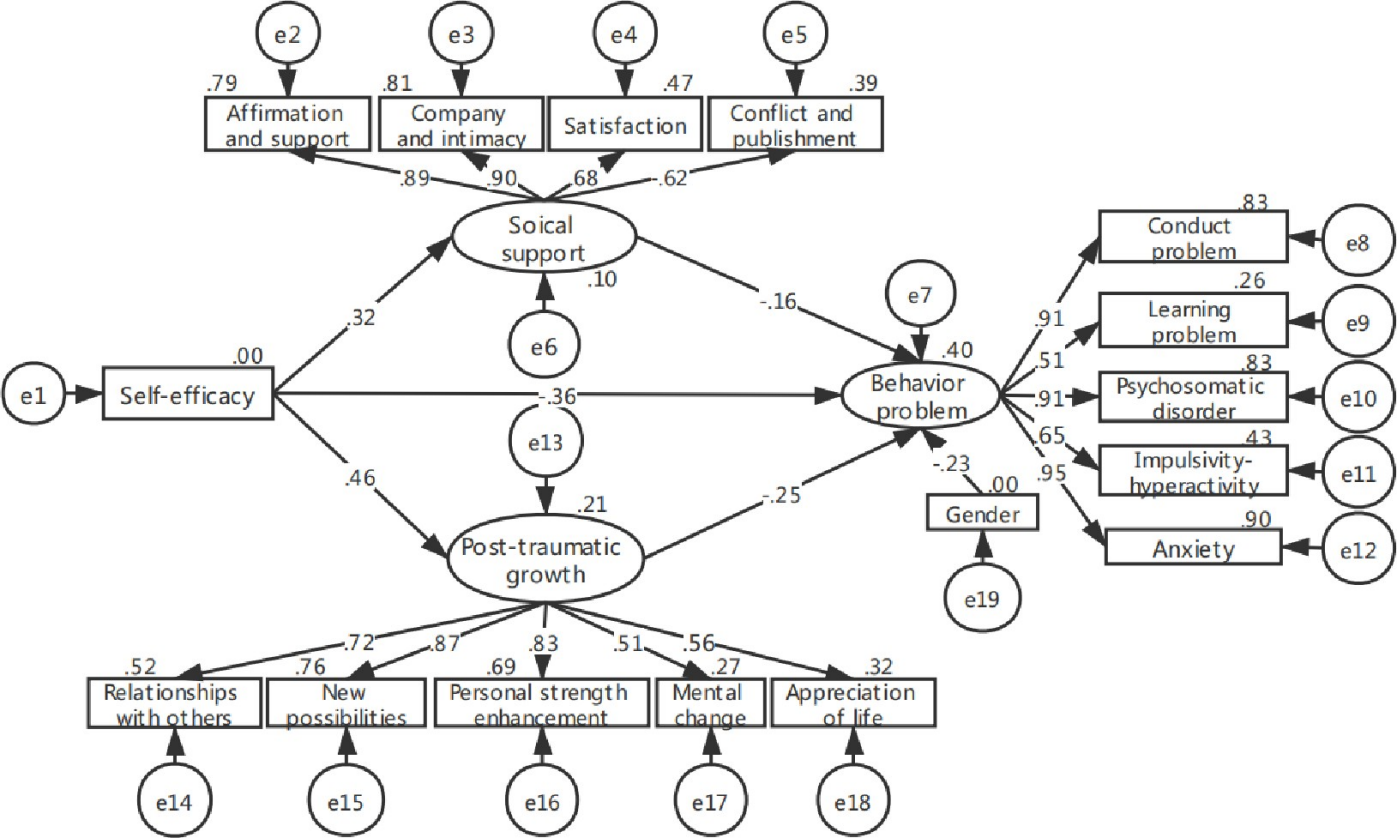
The first model of behavior problems among 8~18 years old children with malignant tumors.

The Kaisser-Meyer-Olkin measure of sampling adequacy (KMO) was .82 and Chi-square of Bartlett’s test was 1482.3 (*P* < .001), which met the basic analysis conditions of the model. After controlling for gender, age ranges and living that were significantly associated with the variablesin factor analyses, the model fit was good (GFI = .87, CFI = .94, AGFI = .87, NFI = .86, χ^2^/df = 1.70, RMSEA = .07). However, the coefficients of the path from age ranges to social support (*P* = .16), the path from living areas to social support (*P* = .09) and the path from living areas to PTG (*P* = .25) were nonsignificant. Thus, the nonsignificant paths were removed to modify the model (every time a parameter was modified or a path was removed, model fitting was performed again), the fitting indexes of the final model (Fig 1) were relatively better (GFI = .92, CFI = .98, AGFI = .87, NFI = .92, χ^2^/df = 1.32, RMSEA = .05).

**Fig 2:**
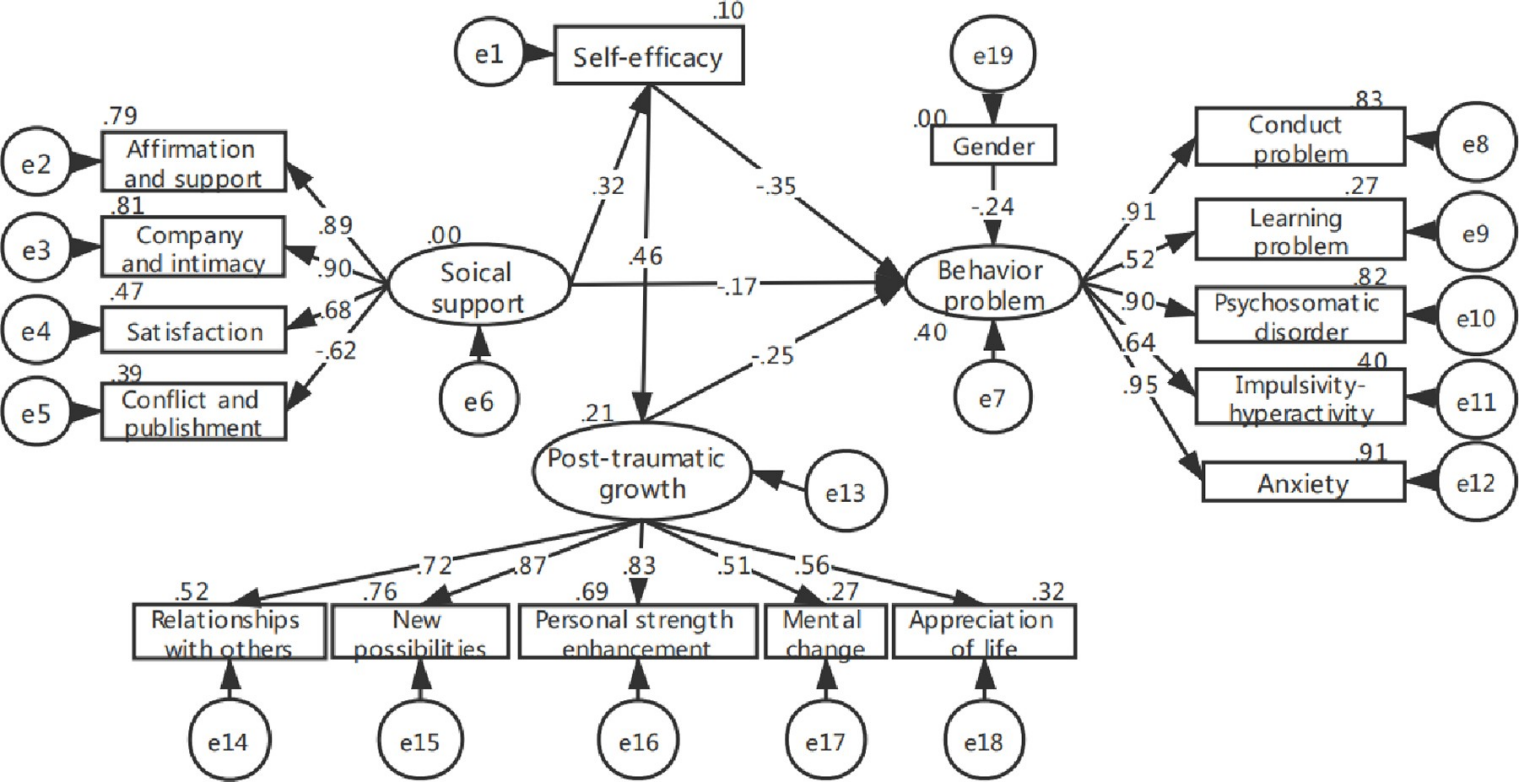
The second model of behavior problems among 8~18 years old children with malignant tumors.

The model shown in Fig 1 only proved that self-efficacy could directly affect social support, however, social support could affect self-efficacy according to previous findings. Thus, in order to explain the influence of social support on self-efficacy, another structural equation model was build after controlling for gender, age ranges and living. The initial model fit was good (GFI = .87, CFI = .94, AGFI = .83, NFI = .86, χ^2^/df = 1.71, RMSEA = .07). However, the coefficients of the path from age ranges to social support (*P* = .19), the path from living areas to social support (*P* = .06), the path from social support to PTG (*P* = .32) and the path from living areas to PTG (*P* = .32) were nonsignificant. Thus, there nonsignificant paths were removed to modify the model (every time a parameter was modified or a path was removed, model fitting was performed again), the final model (Fig 2) fit was relatively better (GFI = .91, CFI = .98, AGFI = .88, NFI = .92, χ^2^/df = 1.29, RMSEA = .04).

As shown in Table 3, girls had less behavior problems than boys, self-efficacy, social support and PTG were negative predictors of 8~18 years old children’s behavior problems. And the standardized total effect of self-efficacy in the first and second model was the largest (*β* = -.525, *P* < .01; *β* = -.468, *P* < .01). Moreover, self-efficacy also indirectly and negatively influenced behavior problems through social support or PTG. Social support could directly affect behavior problems or indirectly predict behavior problems through self-efficacy and PTG.

**Table 3:** Direct, indirect and total effects of variables in the first and second model (N = 160).

Variables	Standardized Direct Effect	Standardized Indirect Effect	Standardized Total Effect
The first model (Fig 1)			
Gender→BP	-.233^a^	—	-.233^a^
SE → BP	-.359^a^	-.166^b^	-.525^a^
SS → BP	-.160^b^	—	-.160^b^
PTG → BP	-.251^a^	—	-.251^a^
The second model (Fig 2)			
Gender→BP	-.235^a^	—	-.235^a^
SS→BP	-.165^b^	-.151^a^	-.317^a^
SE → BP	-.353^a^	-.115^a^	-.468^a^
PTG → BP	-.251^a^	—	-.251^a^

SE: Self-efficacy; SS: Social support; BP: Behavior problem.

^*a*^: *P<*.01;

^*b*^: *P<*.05.

In order to test the mediating effect of self-efficacy, social support and PTG, 2000 bootstrap tests were conducted with Mplus8.3. According to Table 4, 95% confidence intervals (95% CI) of the three mediating pathways didn’t include/cross 0, and the standardized indirect effects are within the confidence intervals, indicating that the mediating effects are significant.

**Table 4:** Bootstrap analysis of mediating effect (N = 160).

Path	Standardized Indirect Effect	95% CI		Ratio
		Lower Limit	Upper Limit	
The first model (Fig 1)				
SE→SS→BP	-.051^b^	-.111	-.014	30.7%
SE→PTG→BP	-.115^a^	-.218	-.069	69.3%
The second model (Fig 2)				
SS→SE→BP	-.114^a^	-.193	-.051	75.5%
SS→SE→PTG→BP	-.037^a^	-.081	-.019	24.5%

SE: Self-efficacy; SS: Social support; BP: Behavior problem.

^a^*P<*.01,

^b^*P<*.05.

## 4. Discussion

A published study showed that the total detection rate of behavior problems in 8~14 years old children with malignant tumors was 24.3% [21]. In this study, the total detection rate of behavior problems in 160 children with malignant tumors in the treatment period was 10.6%, suggesting that behavior problems may be alleviating due to targeted interventions in recent years. However, behavior problems of children with malignant tumors may be more serious than those of normal Chinese children, which partially supported the previous findings that the double hit of early threat and cancer treatments likely alters neural development, and ultimately, cognitive, behavioral, and emotional outcomes [30]. The previous studies confirmed that peer conflict and social withdrawal in children with neuroblastoma were related to gender [31], and attentional problem in children with neurofibromatosis was associated with male gender [32], and the prevalence of any developmental disability increased among boys [33]. Similarly, this study confirmed that boys had more behavior problems than girls, which is probably related to the fact that girls are more encouraged to internalize their feelings and boys are more encouraged to externalize them [34]. Additionally, both of the first model and the second model showed that standardized total effect of self-efficacy on behavior problems was the largest, which demonstrated that self-efficacy played an important role in the occurrence and development of behavior problems in 8~18 years old children with malignant tumors in treatment period. The first model showed that self-efficacy could not only directly affect the behavior problems, but also indirectly predict behavior problems through social support or PTG. The second model proved that social support could directly affect behavior problems or indirectly predict behavior problems through self-efficacy and PTG.

### 4.1. Self-efficacy and its influence on behavior problems

In this study, the mean score of self-efficacy was 14.28, which was lower than the median score of the scale and that of adults with cancer [35]. According to the correlation analysis, the main factor of conduct problems, psychosomatic disorders, impulsivity-hyperactivity and anxiety was self-efficacy, and self-efficacy exerted the greatest impact on impulsivity-hyperactivity (*r* = -.489, *P* < .01). In addition, structural equation model showed that self-efficacy could directly affect behavior problems, which was similar to the previous findings [20,36–37]错误!未定义书签。. It is probable that the children with high self-efficacy are able to confidently manipulate the knowledge or skills they have mastered, concentrate on problem solving and avoid reckless impulsive behavior when facing difficulties or setbacks. Therefore, for low self-efficacy children who emotionally face illness and fear of failure, it is better for medical staff to encourage these children to exchange successful experience with other patients, convince pediatric patients to believe that they own the confidence and capability to overcome difficulties and diseases, thereby improving their self-efficacy level and helping them rationally deal with the negative event like illness.

### 4.2. Social support and its influence on behavior problems

The results showed that the overall social support level of 8~18 years old children with malignant tumors in the treatment period was relatively high in China, which was similar to the findings on the level of social support in adults with cancer [38]. Based on the results of correlation analysis, affirmation and support was the main influencing factor of conduct problems (*r* = -.376, *P* < .01). The reason may be that the family members help solve the problem and encourage children to enhance the sense of belonging, which plays a positive and supportive role in children and helps reduce the occurrence of behavior problems. It can be seen that moderate social support is a protective factor for behavior problems of children with malignant tumors in the treatment period, which expanded on reported findings on the impact of social support on behavior problems [39–40]. At present, Chinese parents don't pay much attention to the cultivation and education of the good behavior habits for the only child. Parent’s over-protection will cause children to become self-centered, grumpy and lead to other behavior problems. Different from this, the education of American families is mainly embodied in independence and democracy, i.e., parents pay attention to the cultivation of children's subjective initiative and the whole family advocates democracy. However, excessive democratic education also makes a great number of children to be self-centered and stubborn. Therefore, medical staff should combine the advantages of domestic and foreign education to guide family members to treat children equally, communicate and educate their children in time. Moreover, understanding and patience can help children face the disease positively, so as to prevent or reduce the occurrence of conduct problems such as denying mistakes, being rude and quarreling.

### 4.3. PTG and its influence on behavior problems

In this study, the total PTG score of 160 Chinese children with malignant tumors during the treatment was (41.64±15.3), which was lower than that of adults with cancers domestically and abroad [41–42], indicating that although 8~18 years old children with malignant tumors gained a certain degree of growth, the overall level was low, which may be related to their immature mind or incomplete cognitive development. Furthermore, it was found that there was a significant positive correlation between PTG and behavior problems of children with malignant tumors in the treatment period, and the correlation analysis showed that new possibility dimension had the greatest impact on the anxiety dimension (*r* = -.387, *P* < .01). Positive post-traumatic changes, including developing new hobbies, owning new opportunities and establishing new directions of life, could reduce the negative perception of disease and the sensitivity to negative emotions, promote children to focus on positive self-development, which is helpful to reduce the occurrence of anxiety. Thus, parents can ask teachers or their children’s classmates to communicate with the child frequently, which is conducive to the positive growth after trauma [43]. Meanwhile, medical staff can help children look for hobbies and encourage them to participate in the activities organized by department. Besides, based on the evaluation of children's cognition, children's mild cognitive distortion can be corrected through cognitive reconstruction. For children with moderate or severe cognitive distortion, professional psychological consultants should be involved to help children actively deal with the disease, and then improve the level of PTG and ameliorate behavior problems [44–45].

### 4.4. Partial mediation of self-efficacy, social support and PTG

The first model showed that self-efficacy not only directly predicted behavior problems of children with malignant tumors during the treatment period, but also indirectly affected the behavior problems through social support and PTG. This finding confirmed that children with high-level self-efficacy may gain more support and obtain a higher level of PTG, which was beneficial to reduce the occurrence of behavior problems. The second model showed that social support could indirectly predict behavior problems through self-efficacy and PTG, meaning that getting more social support helps to improve self-efficacy and PTG, which was good for behavior problems.

The explanation of these findings may be that individuals with high self-efficacy can deal with difficulties and setbacks rationally, control the idea of self-abandonment and tend to meet challenges optimistically, which helps achieve more positive PTG. Furthermore, social support provides supportive environment for individuals after trauma and help improve the level of self-efficacy, which is beneficial to think about the positive significance of traumatic events. Thus, parents should teach pediatric patients to independently complete daily chores to increase their successful experience and self-efficacy. As one of the individuals who can provide social support, medical staff can say some encouraging words to children, so as to effectively improve the self-efficacy and promote the PTG of children with malignant tumors in the treatment period. Moreover, the department can cooperate with volunteers to establish a family-centered care team, regularly carry out hospitalization or community activities, encourage children to share and express with each other. A long-term and effective support system can be established to improve child behavior problems.

## 5. Strengths and limitations

This is the first study to explore the influence of self-efficacy, social support and PTG on behavior problems of Chinese children with malignant tumors, which provides a reference for preventing or reducing the occurrence of child behavior problems. However, this study has some limitations. This study is a cross-sectional study design and lacks the data of time interval between the beginning of treatment and data collection, so longitudinal research is needed to examine how these variables change over time and the data of time interval should be collected. Some published studies have found the influence of cognitive abilities on behavior problem, but this study can’t confirm the correlation between cognitive ability and behavior problem because we only roughly judge the child's cognitive ability through communication or daily observation. Thus, it’s necessary to perform the psychometric cognitive assessment by professional clinical assessment tools or psychological interviews. Additionally, this study didn’t use a self-reported measure of behavior problems and absented the validated measures with referenced standard scores. Thus, we will consider using a self-report measure in the future research protocols and adding the validated measures with referenced standard scores to confirm the findings of this study.

## 6. Conclusion

This study contributes to a better understanding of the determinants of behavior problem in children with malignant tumors during treatment. Self-efficacy was directly and negatively correlated with child behavior problems. Social support and PTG mediated the relationship between self-efficacy and behavior problems, demonstrating that children with lower-level self-efficacy may have less social support and PTG, leading to more behavior problems. Self-efficacy and PTG mediated the relationship between social support and behavior problems, suggesting that children with lower-level social support may have lower-level self-efficacy and PTG, resulting in more behavior problems. Therefore, focusing on strengthening the self-efficacy, social support and PTG of children with malignant tumors may positively affect their behavior problems.

## Supporting information

S1 Table Sample characteristics and behavior problems (N = 160). “Others” includes immature teratoma, dysgerminoma, yolk sac tumour of the ovary, neuroblastoma and hepatoblastoma.
(DOCX)

S2 Table Factor analysis(N = 160). “Others” includes immature teratoma, dysgerminoma, yolk sac tumour of the ovary, neuroblastoma and hepatoblastoma. CP: conduct problem; LP: learning problem; PD: psychosomatic disorders; IH: impulsivity-hyperactivity; An: anxiety; SE:self-efficacy; AS: affirmation and support; CI: company and intimacy; Sa: satisfaction; Cp:conflict and publishment; RO: relationship with others; NP: new possibilities; PE: personal strength enhancement; MC: mental change; AL: appreciation of life.
(DOCX)

S3 Table Correlation between observed indicators of latent variables (N = 160). (DOCX)

S4 Table Direct, indirect and total effects of variables in the first and second model (N = 160). SE: Self-efficacy; SS: Social support; BP: Behavior problem.
(DOCX)

S5 Table Bootstrap analysis of mediating effect (N = 160). SE: Self-efficacy; SS: Social support; BP: Behavior problem.
(DOCX)

S1 Fig The first model of behavior problems among 8~18 years old children with malignant tumors. (TIF)

S2 Fig The second model of behavior problems among 8~18 years old children with malignant tumors. (TIF)

S1 Checklist (DOCX)
