# Correction: The effects of self-efficacy and social support on behavior problems in 8~18 years old children with malignant tumors

**DOI:** 10.1371/journal.pone.0251941

**Published:** 2021-05-13

**Authors:** Qian Liu, Lin Mo, Xianqiao Huang, Lu Yu, Yang Liu

None of the authors should have been attributed equal contribution to this work.
