# Supplementary material for: The effects of self-efficacy and social support on behavior problems in 8~18 years old children with malignant tumors
Source: PLoS One. 2020 Jul 31;15(7):e0236648. doi: 10.1371/journal.pone.0236648 (PMC7394414; doi:10.1371/journal.pone.0236648)
Supplement: S1 Table — “Others” includes immature teratoma, dysgerminoma, yolk sac tumour of the ovary, neuroblastoma and hepatoblastoma. (DOCX) [file pone.0236648.s001.docx]

**Table 1. Sample characteristics and behavior problems ( (N=160).**

| Characteristics | n (%) | $\overline{\text{X}}$±SD |
| --- | --- | --- |
| Gender |  |  |
| boy | 93(58.1) |  |
| girl | 67(41.9) |  |
| Age range (years) |  |  |
| 8~12 | 107(66.9) |  |
| 13~18 | 53(33.1) |  |
| Type of disease |  |  |
| leukemia | 94(58.8) |  |
| lymphoma | 27(16.9) |  |
| sarcoma | 17(10.6) |  |
| others | 22(13.8) |  |
| Living area |  |  |
| rural | 113(70.6) |  |
| urban | 47(29.4) |  |
| Self-efficacy |  | 14.3±4.2 |
| low-level | 124(77.5) | 12.6±2.8 |
| medium-level | 32(20.0) | 19.6±1.9 |
| high-level | 4(2.5) | 24.5±0.6 |
| Social support |  |  |
| affirmation and support |  | 142.5±29.4 |
| company and intimacy |  | 98.8±21.5 |
| satisfaction |  | 62.1±11.2 |
| conflict and publishment |  | 82.0±19.0 |
| Post-traumatic growth |  | 41.6±15.3 |
| relationship with others |  | 14.1±5.3 |
| new possibilities |  | 9.3±4.8 |
| personal strength enhancement |  | 8.0±4.0 |
| mental change |  | 2.8±2.0 |
| appreciation of life |  | 7.8±3.3 |
| Behavior problem |  |  |
| conduct problems |  | .53±.34 |
| learning problems |  | .79±.51 |
| psychosomatic disorders |  | .41±.35 |
| impulsivity-hyperactivity |  | .53±.48 |
| anxiety |  | .49±.42 |

Note: “Others” includes immature teratoma, dysgerminoma, yolk sac tumour of the ovary, neuroblastoma and hepatoblastoma.
