# Supplementary material for: The effects of self-efficacy and social support on behavior problems in 8~18 years old children with malignant tumors
Source: PLoS One. 2020 Jul 31;15(7):e0236648. doi: 10.1371/journal.pone.0236648 (PMC7394414; doi:10.1371/journal.pone.0236648)
Supplement: S2 Table — “Others” includes immature teratoma, dysgerminoma, yolk sac tumour of the ovary, neuroblastoma and hepatoblastoma. CP: conduct problem; LP: learning problem; PD: psychosomatic disorders; IH: impulsivity-hyperactivity; An: anxiety; SE:self-efficacy; AS: affirmation and support; CI: company and intimacy; Sa: satisfaction; Cp:conflict and publishment; RO: relationship with others; NP: new possibilities; PE: personal strength enhancement; MC: mental change; AL: appreciation of life. (DOCX) [file pone.0236648.s002.docx]

**Table 2. Factor analysis(N=160).**

| Variables | Behavior problem | | | | | | | | | | SE | *t*/*F* | Social support | | | | | | | | Post-traumatic growth | | | | | | | | | |
| --- | --- | --- | --- | --- | --- | --- | --- | --- | --- | --- | --- | --- | --- | --- | --- | --- | --- | --- | --- | --- | --- | --- | --- | --- | --- | --- | --- | --- | --- | --- |
|  | CP | *t*/*F* | LP | *t*/*F* | PD | *t*/*F* | IH | *t*/*F* | An | *t*/*F* |  |  | AS | *t*/*F* | CI | *t*/*F* | Sa | *t*/*F* | Cp | *t*/*F* | RO | *t*/*F* | *NP* | *t*/*F* | PE | *t*/*F* | MC | *t*/*F* | AL | *t*/*F* |
| Gender |  |  |  |  |  |  |  |  |  |  |  |  |  |  |  |  |  |  |  |  |  |  |  |  |  |  |  |  |  |  |
| boy | .6±.4 | 1.8 | .9±.5 | 2.3^b^ | .5±.4 | 2.0 | .5±.5 | .4 | .6±.5 | 2.3^b^ | 14.1±4.4 | -.6 | 141.8±30.5 | -.4 | 100.3±24.0 | 1.1 | 61.2±12.1 | -1.2 | 82.6±18.1 | .5 | 14.4±5.5 | 1.0 | 9.8±5.0 | 1.8 | 8.4±4.3 | 1.7 | 2.8±2.0 | .5 | 7.9±3.5 | .9 |
| girl | .5±.3 |  | .7±.5 |  | .4±.3 |  | .5±.5 |  | .4±.3 |  | 14.5±3.9 |  | 143.6±27.9 |  | 96.6±17.1 |  | 63.4±9.7 |  | 81.1±20.3 |  | 13.6±5.1 |  | 8.5±4.4 |  | 7.3±3.4 |  | 2.7±2.0 |  | 7.5±2.8 |  |
| Age range  (years) |  |  |  |  |  |  |  |  |  |  |  |  |  |  |  |  |  |  |  |  |  |  |  |  |  |  |  |  |  |  |
| 8~12 | .5±.3 | .2 | .8±.5 | .2 | .4±.4 | .4 | .5±.5 | .1 | .5±.4 | .3 | 14.3±4.2 | .0 | 143.1±29.5 | .3 | 101.2±21.9 | 2.1^b^ | 62.7±11.3 | .9 | 79.7±17.5 | -2.0^b^ | 14.2±5.7 | .5 | 9.5±5.2 | 1.0 | 8.1±4.3 | .9 | 2.8±1.9 | .0 | 7.7±3.3 | -.4 |
| 13~18 | .5±.4 |  | .8±.5 |  | .4±.4 |  | .5±5 |  | .5±.4 |  | 14.3±4.0 |  | 141.5±29.4 |  | 93.9±19.9 |  | 61.0±10.9 |  | 86.5±21.1 |  | 13.8±4.7 |  | 8.7±3.8 |  | 7.6±3.3 |  | 2.8±2.2 |  | 7.9±3.2 |  |
| Type of disease |  |  |  |  |  |  |  |  |  |  |  |  |  |  |  |  |  |  |  |  |  |  |  |  |  |  |  |  |  |  |
| leukemia | .5±.3 | .9 | .8±.5 | .7 | .4±.3 | .8 | .5±.5 | 1.2 | .5±.4 | .3 | 14.6±4.2 | .6 | 143.2±29.6 | .5 | 99.6±23.0 | .8 | 62.7±11.2 | .2 | 82.5±19.6 | .9 | 13.5±4.9 | 1.1 | 9.2±4.8 | .2 | 7.8±4.0 | .6 | 2.8±2.1 | 1.3 | 7.6±3.3 | .8 |
| lymphoma | .6±.4 |  | .7±.5 |  | .4±.4 |  | .5±.5 |  | .5±.4 |  | 13.9±4.5 |  | 136.7±28.8 |  | 93.0±19.9 |  | 61.1±13.3 |  | 85.1±18.0 |  | 14.9±6.5 |  | 9.8±4.9 |  | 8.5±4.2 |  | 3.2±2.0 |  | 8.4±3.5 |  |
| sarcoma | .4±.3 |  | 1.0±.5 |  | .4±.3 |  | .5±5 |  | .5±.4 |  | 13.4±3.2 |  | 144.5±23.3 |  | 100.2±18.4 |  | 61.8±11.6 |  | 75.6±17.7 |  | 15.8±5.9 |  | 9.4±4.7 |  | 8.8±4.4 |  | 2.8±2.0 |  | 6.9±3.0 |  |
| others | .6±.4 |  | .8±.7 |  | .5±.5 |  | .7±.5 |  | .6±.6 |  | 14.0±4.5 |  | 145.4±33.8 |  | 101.3±18.8 |  | 61.2±8.3 |  | 81.0±18.5 |  | 14.1±4.9 |  | 8.8±5.1 |  | 7.4±3.5 |  | 2.0±1.8 |  | 8.0±3.1 |  |
| Living area |  |  |  |  |  |  |  |  |  |  |  |  |  |  |  |  |  |  |  |  |  |  |  |  |  |  |  |  |  |  |
| rural | .5±.3 | -.8 | .7±.5 | -1.8 | .4±.4 | -.6 | .5±.5 | -1.3 | .5±.4 | -.6 | 14.5±4.2 | .9 | 144.7±29.2 | 1.5 | 100.7±21.2 | 1.8 | 63.0±11.3 | 1.5 | 79.5±17.7 | -2.5^b^ | 14.6±5.6 | 2.3^a^ | 9.7±5.0 | 1.7 | 8.1±4.3 | .7 | 2.7±2.0 | -.7 | 7.9±3.3 | .8 |
| urban | .7±.5 |  | .9±.6 |  | .4±.4 |  | .6±.5 |  | .5±.4 |  | 13.9±4.0 |  | 137.2±29.3 |  | 94.1±21.7 |  | 60.1±10.9 |  | 88.0±20.8 |  | 12.7±4.3 |  | 8.3±4.1 |  | 7.6±3.3 |  | 2.9±2.1 |  | 7.4±3.2 |  |

Note1. “Others” includes immature teratoma, dysgerminoma, yolk sac tumour of the ovary, neuroblastoma and hepatoblastoma.

Note2. CP: conduct problem; LP: learning problem; PD: psychosomatic disorders; IH: impulsivity-hyperactivity; An: anxiety; SE:self-efficacy; AS: affirmation and support; CI: company and intimacy; Sa: satisfaction; Cp:conflict and publishment; RO: relationship with others; NP: new possibilities; PE: personal strength enhancement; MC: mental change; AL: appreciation of life.

Note3. *^a^*: *P*<.01; *^b^*: *P*<.05.
