# Supplementary material for: The effects of self-efficacy and social support on behavior problems in 8~18 years old children with malignant tumors
Source: PLoS One. 2020 Jul 31;15(7):e0236648. doi: 10.1371/journal.pone.0236648 (PMC7394414; doi:10.1371/journal.pone.0236648)
Supplement: S3 Table — (DOCX) [file pone.0236648.s003.docx]

**Table 3. Correlation between Observed Indicators of Latent Variables (N=160).**

| Variables | Correlations between Variables | | | | | | | | | | | | | | |
| --- | --- | --- | --- | --- | --- | --- | --- | --- | --- | --- | --- | --- | --- | --- | --- |
|  | 1 | 2 | 3 | 4 | 5 | 6 | 7 | 8 | 9 | 10 | 11 | 12 | 13 | 14 | 15 |
| 1 Self-efficacy | 1 | — | — | — | — | — | — | — | — | — | — | — | — | — | — |
| Social support |  |  |  |  |  |  |  |  |  |  |  |  |  |  |  |
| 2 affirmation and support | .30^a^ | 1 | — | — | — | — | — | — | — | — | — | — | — | — | — |
| 3 company and intimacy | .31^a^ | .80^a^ | 1 | — | — | — | — | — | — | — | — | — | — | — | — |
| 4 satisfaction | .13 | .63^a^ | .61^a^ | 1 | — | — | — | — | — | — | — | — | — | — | — |
| 5 conflict and publishment | -.10 | -.54^a^ | -.59^a^ | -.59^a^ | 1 | — | — | — | — | — | — | — | — | — | — |
| Post-traumatic growth |  |  |  |  |  |  |  |  |  |  |  |  |  |  |  |
| 6 relationships with others | .29^a^ | .25^a^ | .21^a^ | .11 | -.11 | 1 | — | — | — | — | — | — | — | — | — |
| 7 new possibilities | .45^a^ | .20^b^ | .21^a^ | .10 | -.11 | .61^a^ | 1 | — | — | — | — | — | — | — | — |
| 8 personal strength enhancement | .33^a^ | .18^b^ | .20^b^ | .10 | .05 | .63^a^ | .72^a^ | 1 | — | — | — | — | — | — | — |
| 9 mental change | .28^a^ | .06 | -.01 | -.11 | .01 | .34^a^ | .48^a^ | .38^a^ | 1 | — | — | — | — | — | — |
| 10 appreciation of life | .24^a^ | .05 | .00 | .02 | -.02 | .46^a^ | .46^a^ | .48^a^ | .27^a^ | 1 | — | — | — | — | — |
| Behavior problem |  |  |  |  |  |  |  |  |  |  |  |  |  |  |  |
| 11 conduct problems | -.45^a^ | -.38^a^ | -.34^a^ | -.21^a^ | .19^b^ | -.20^a^ | -.33^a^ | -.26^a^ | -.25^a^ | -.21^a^ | 1 | — | — | — | — |
| 12 learning problems | -.28^a^ | -.24^a^ | .18^b^ | -.15 | .06 | -.27^a^ | -.24^a^ | -.29^a^ | -.17^b^ | -.29^a^ | .46^a^ | 1 | — | — | — |
| 13 Psychosomatic disorders | -.48^a^ | -.33^a^ | -.27^a^ | -.15 | .11 | -.27^a^ | -.34^a^ | -.28^a^ | -.29^a^ | -.25^a^ | .82^a^ | .58^a^ | 1 | — | — |
| 14 impulsivity-hyperactivity | -.49^a^ | -.17^a^ | -.16^b^ | -.08 | .08 | -.27^a^ | -.39^a^ | -.27^a^ | -.25^a^ | -.29^a^ | .51^a^ | .30^a^ | .62^a^ | 1 | — |
| 15 anxiety | -.47^a^ | -.33^a^ | -.28^a^ | -.14 | .12 | -.30^a^ | -.39^a^ | -.36^a^ | -.29^a^ | -.28^a^ | .86^a^ | .70^a^ | .85^a^ | .60^a^ | 1 |

Note1. *^a^*: *P*<.01; *^b^*: *P*<.05.
