# Supplementary material for: The effects of self-efficacy and social support on behavior problems in 8~18 years old children with malignant tumors
Source: PLoS One. 2020 Jul 31;15(7):e0236648. doi: 10.1371/journal.pone.0236648 (PMC7394414; doi:10.1371/journal.pone.0236648)
Supplement: S4 Table — SE: Self-efficacy; SS: Social support; BP: Behavior problem. (DOCX) [file pone.0236648.s004.docx]

**Table 4. Direct, Indirect and Total Effects of Variables in the First and Second Model (N=160).**

| Variables | Standardized Direct Effect | Standardized Indirect Effect | Standardized Total Effect |
| --- | --- | --- | --- |
| The first model(Fig 1) | | | |
| Gender→BP | -.233^a^ | — | -.233^a^ |
| SE → BP | -.359^a^ | -.166^b^ | -.525^a^ |
| SS → BP | -.160^b^ | — | -.160^b^ |
| PTG → BP | -.251^a^ | — | -.251^a^ |
| The second model(Fig 2) | | | |
| Gender→BP | -.235^a^ | — | -.235^a^ |
| SS→BP | -.165^b^ | -.151^a^ | -.317^a^ |
| SE → BP | -.353^a^ | -.115^a^ | -.468^a^ |
| PTG → BP | -.251^a^ | — | -.251^a^ |

Note1. SE: Self-efficacy; SS: Social support; BP: Behavior problem.

Note2. *^a^*: *P*＜.01; *^b^*: *P*＜.05..
