# Supplementary material for: The effects of self-efficacy and social support on behavior problems in 8~18 years old children with malignant tumors
Source: PLoS One. 2020 Jul 31;15(7):e0236648. doi: 10.1371/journal.pone.0236648 (PMC7394414; doi:10.1371/journal.pone.0236648)
Supplement: S5 Table — SE: Self-efficacy; SS: Social support; BP: Behavior problem. (DOCX) [file pone.0236648.s005.docx]

**Table 5. Bootstrap Analysis of Mediating Effect (N=160).**

| Path | Standardized Indirect Effect | 95% CI | | Ratio |
| --- | --- | --- | --- | --- |
|  |  | Lower Limit | Upper Limit |  |
| The first model(Fig 1) | | | | |
| SE→SS→BP | -.051^b^ | -.111 | -.014 | 30.7% |
| SE→PTG→BP | -.115^a^ | -.218 | -.069 | 69.3% |
| The second model(Fig 2) | | | | |
| SS→SE→BP | -.114^a^ | -.193 | -.051 | 75.5% |
| SS→SE→PTG→BP | -.037^a^ | -.081 | -.019 | 24.5% |

Note1. SE: Self-efficacy; SS: Social support; BP: Behavior problem.

Note2. ^a^*P*＜.01, ^b^*P*＜.05.
