# Supplementary figures and images for: The effects of self-efficacy and social support on behavior problems in 8~18 years old children with malignant tumors
Source: PLoS One. 2020 Jul 31;15(7):e0236648. doi: 10.1371/journal.pone.0236648 (PMC7394414; doi:10.1371/journal.pone.0236648)

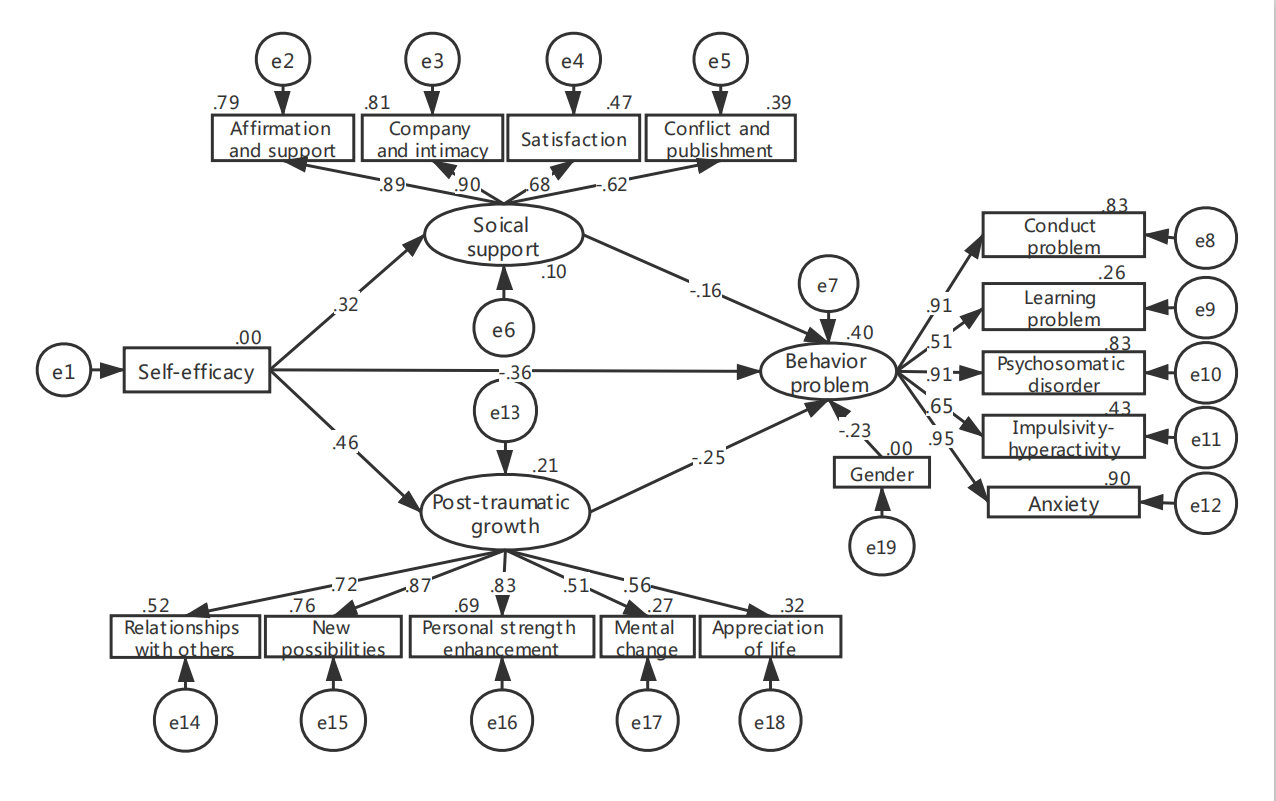

Supplement: S1 Fig — (TIF) [file pone.0236648.s006.tif]

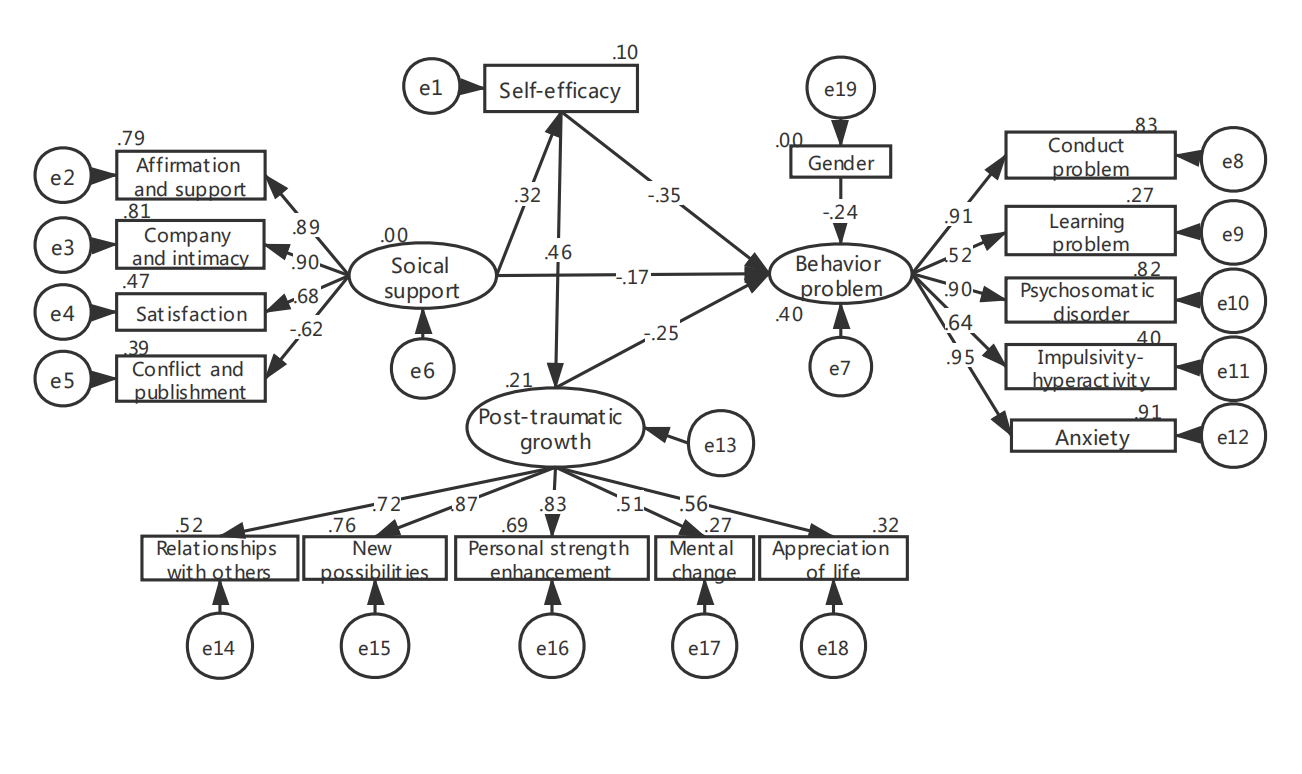

Supplement: S2 Fig — (TIF) [file pone.0236648.s007.tif]
